# Wheat-Related Gastrointestinal Diseases: Narrative Review

**DOI:** 10.5152/tjg.2025.25375

**Published:** 2025-09-01

**Authors:** Umael Khan, Gülen Arslan Lied

**Affiliations:** 1 Section of Gastroenterology, Department of Medicine, Haukeland University Hospital, Bergen, Norway; 2 National Centre for Functional Gastrointestinal Disorders, Haukeland University Hospital, Bergen, Norway; 3 Centre for Nutrition, Department of Clinical Medicine, University of Bergen, Norway

**Keywords:** Celiac disease, diet, exercise-induced allergies, food hypersensitivity, gluten-free, glutens, irritable bowel syndrome, wheat hypersensitivity

## Abstract

Intolerance to wheat and gluten intake has gained public and scientific interest in recent years. Celiac disease (CD) and wheat allergy are wheat-related disorders with a well-defined etiological mechanism alongside corresponding diagnostic tests. In addition, patients also self-report intolerance toward wheat and gluten that does not meet the criteria of CD and wheat allergy. This gives rise to a third category, namely non-celiac wheat sensitivity (also referred to as non-celiac gluten sensitivity). However, this category is controversial. Unlike CD and wheat allergy, the pathophysiological mechanism is unknown. When conducting double-blinded placebo-controlled trials, only a small proportion of patients can correctly identify gluten from a placebo based on symptoms, indicating a substantial nocebo component. In fact, it has been posited that non-celiac wheat sensitivity is simply a form of irritable bowel syndrome. The aim of this review is to provide an overview of the epidemiology, etiology, clinical manifestations, diagnosis, and management of the 3 abovementioned conditions.

**Main Points**

- Wheat-related food intolerance can be divided into 3 main categories: celiac disease (CD), wheat allergy, and non-celiac wheat/gluten sensitivity.
- Whereas CD and wheat allergy are wheat-related disorders with a well-defined mechanism, non-celiac wheat/gluten sensitivity remains more controversial.
- The distinction between non-celiac wheat/gluten sensitivity and irritable bowel syndrome also remains controversial.

## Introduction

**Figure 1.**
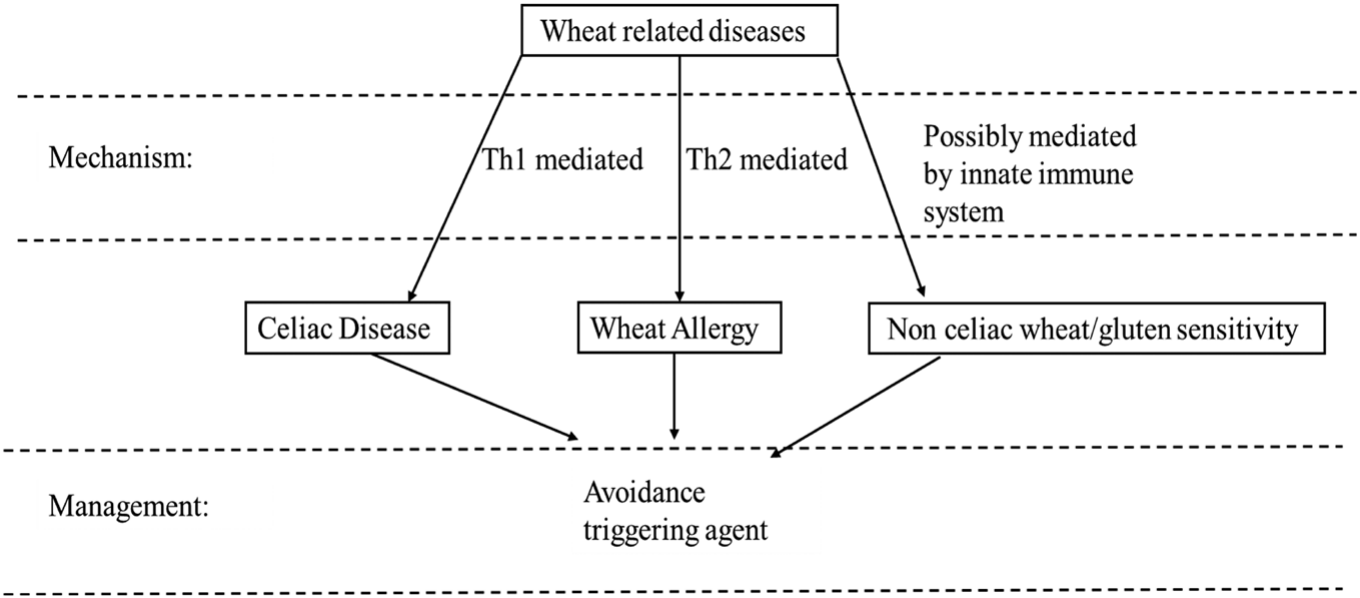
Wheat-related diseases. The figure presents an overview of wheat-related disorders, their respective pathogenesis, as well as the convergence of treatment centered around the avoidance of the offending agent.

Alongside maize and rice, wheat constitutes a major staple crop for a significant proportion of the global population.^1^ In recent years, gluten-free diets have gained traction due to more self-diagnosed wheat intolerance.^2^ This increased public interest in wheat intolerance presents physicians, nutritionists, and public health professionals with both opportunities and challenges. On one hand, celiac disease (CD) and wheat allergy are disorders where both the etiology and the benefit of wheat avoidance are well documented;^3^ more effective case finding could benefit more patients and significantly improve quality of life. Conversely, wheat intolerance that does not meet the criteria for CD or wheat allergy, namely non-celiac wheat/gluten sensitivity (NCWS/NCGS), remains more controversial. The aim of this review is to present an overview of the abovementioned conditions. Figure 1 presents an overview of these conditions.

### Wheat Proteins

Wheat proteins comprise approximately 10%-15% of the total wheat dry weight.^4^ They are the primary drivers of both CD and wheat allergy. These proteins can be classified according to salt solubility. There is a salt-soluble fraction, which includes amylase-trypsin inhibitor subunits as well as other proteins such as lipid transfer proteins.^4^ Conversely, there is a salt-insoluble fraction that includes gluten, which can be subdivided into glutenins and gliadins.^4^ Gliadins, in turn, are further subdivided into α-, β-, γ-, and ω-gliadins, whereas glutenins are further subdivided into high-molecular-weight and low-molecular-weight glutenins.^2^

## Celiac Disease

### Epidemiology

The global prevalence of CD is approximately 1%.^5,6^ While often thought to be primarily associated with northwestern European ancestry, the highest prevalence is actually found within the Saharawi population (5.6%);^7^ the prevalence in low-income countries is likely underestimated due to a lack of diagnostic infrastructure and experience.^7-9^ The overarching trend is toward an increased prevalence of CD. While this could be partly explained by an increased testing rate, this is likely not the entire explanation; a large population-based Canadian cohort study found that even in the case of a stable testing rate, there is still a rising incidence of CD.^10^

### Etiology

Celiac disease is due to a T-cell–mediated adverse response to gluten intake.^11^ Following gluten intake, the gliadin component is deamidated by tissue transglutaminase 2 (TG2), converting its glutamine into glutamic acid. This deamidation increases binding affinity to HLA DQ2 or HLA DQ8 on antigen-presenting cells, which in turn leads to activation of intestinal T-cells that target the intestinal epithelium. This process is facilitated by Th1 activation.^12^ Histopathological assessment reveals leukocyte infiltration, regenerative crypt hyperplasia, and villous atrophy of intestinal epithelial cells; the extent of these changes forms the basis of histopathological classification schemes such as Marsh and Corazza-Villanacci.^13^

Genetic predisposition plays a significant role in the development of CD. This is illustrated by twin studies that reveal higher concordance in monozygotic twins compared to dizygotic twins.^14^ Most patients have either HLA class II heterodimer subtypes HLA DQ2 or HLA DQ8. Due to the high prevalence of these haplotypes in healthy subjects,^15^ their presence does not confer a high positive predictive value. However, their absence renders CD highly unlikely. It should also be noted, though, that a small minority of celiac patients carry the HLA D7 haplotype.^16-18^ In addition to HLA haplotyping, other genetic loci associated with CD have also been identified.^19^

Beyond gluten, the role of other environmental factors is less understood. A Danish cohort study found that viral croup and otitis media by 18 months of age increased the risk of later development of CD.^20^ A meta-analysis found that early (4-6 months) compared to late (after 6 months) introduction of gluten in the diet increased the risk of CD.^21^ The same paper did not find breastfeeding to affect the development of CD.^21^ Gastrointestinal infections, surgery, and medications have also been implicated as contributing agents of CD.^22-24^ The role of genetic and environmental factors in the development of CD remains an area of ongoing research.

### Clinical Manifestations

There is a wide range of clinical symptoms associated with CD. In a recent cohort study, 51% of patients reported gastrointestinal symptoms, with bloating in 28%, diarrhea in 24.3%, and aphthous stomatitis in 19.7%.^25^ Extraintestinal manifestations included anemia (35.8% iron-deficiency; 87% folic acid malabsorption), cryptogenic hypertransaminasemia (27.9%), and recurrent miscarriages (11.5%).^25^ Extraintestinal manifestations include, but are not limited to, gluten ataxia, peripheral neuropathy, gluten-induced hepatitis, dermatitis herpetiformis, and enamel defects.^26^ A detailed description of these manifestations and their management is beyond the scope of this review.

### Diagnosis

Neither the 2019 guidelines from the European Society for the Study of Celiac Disease (ESsCD) nor the 2023 American College of Gastroenterology (ACG) guidelines recommended population screening.^3,27^ Rather, active case-finding (testing for CD among individuals with symptoms, including subtle or atypical symptoms, and in high-risk groups such as first-degree relatives of CD patients) is recommended instead.

For adult diagnostics, a combination of serological testing and duodenal biopsies while on a gluten-containing diet is recommended.^3,27^ With regard to serology, the current benchmark is autoantibodies to the mucosal enzyme TG2. A Norwegian population study published in 2025 strengthens the role of IgA TG2, showing it to have excellent diagnostic accuracy with regard to CD.^28^ Positive IgA TG2 warrants confirmation with duodenal biopsies. At least 4 duodenal biopsies should be acquired from the descending duodenum.^3,27^ In addition, it is also recommended to acquire bulbar biopsies.^3,27^ With regard to dermatitis herpetiformis and gluten ataxia, details regarding diagnosis are presented elsewhere.^29,30^

According to the 2020 guidelines from the European Society of Pediatric Gastroenterology, Hepatology, and Nutrition, CD may be diagnosed in children without the use of a biopsy. It requires IgA TG2 to be >10x upper limits of normal, supplemented with positive endomysial antibodies (EMA) in a separate blood sample.^31^ Due to a paucity of studies verifying this approach in adults, neither the ACG nor ESsCD recommends this approach in adults.^3,27^ However, the 2023 ACG guidelines allow for the consideration of this approach in patients unable or unwilling to undergo a gastroscopy with duodenal biopsies.^27^

The combination of serology and biopsies does not always yield clear answers. In the case of positive serology and normal histology (provided there is HLA-DQ2/8), the patient is regarded as having potential CD.^3,27^ Differential diagnoses such as hypergammaglobulinemia, autoimmune diseases, chronic liver disease, congestive heart failure, and enteric infections have shown false-positive results and should be considered. On the other hand, the presence of other antibodies, such as EMA, would strengthen the diagnosis.^3,27^ Duodenal lymphocytosis without villous atrophy is also a non-specific finding and can be seen in cases of inflammatory bowel disease, eosinophilic enteritis, food allergies, autoimmune enteritis, common variable immune deficiency, peptic injury, and infections.^32^ The EScCD guidelines provide recommendations on the assessment of duodenal lymphocytosis.^3^ If both TTG2 and EMA are positive, CD is the most likely diagnosis, and a gluten-free diet can be attempted with a reassessment of serology and biopsies after a year. If only TTG2 is positive, one may assess for the presence of HLA DQ2/8. If HLA DQ2/8 is absent, CD is unlikely. If it is positive, it is recommended to reassess serology after 6-12 months, and a new biopsy may be considered. Finally, the case of negative serology with villous atrophy also posits a diagnostic challenge. A range of non-celiac enteropathies can present with villous atrophy. These include infections, drugs, and malignancy.^33^ At the same time, CD can also present with negative serology, with the proportion of seronegative CD being estimated at 1.7%-5%.^34^ Details surrounding the diagnostic approach to seronegative CD and other seronegative enteropathies can be found in the American Gastroenterological Association clinical practice update on seronegative enteropathies^34^ as well as the EScCD guidelines.^3^

### Management

The cornerstone of CD treatment is a lifelong gluten-free diet, which entails the exclusion of wheat, barley, and rye due to their prolamine components (gliadins, secalins, and hordeins, respectively).^3^ While the prolamine component in oats (avenins) is usually well tolerated,^35^ there are conflicting reports on this matter, so monitoring for oat tolerance is recommended.^27^ Micronutrient screening is also recommended, including iron,^36^ folate,^37^ vitamin B6,^38^ B12,^37^ copper,^39^ magnesium,^40^ and zinc.^40^ In addition, regular osteoporosis screening should be performed, depending on the age of presentation as well as risk factors.^3^ Macronutrient imbalance is also a challenge in CD patients undergoing a gluten-free diet; a study examining fatty liver disease as well as metabolic syndrome found that CD patients are at high risk for developing metabolic syndrome as well as fatty liver, with this risk increasing further following a gluten-free diet.^41^ It is therefore recommended that a nutritionist also be involved in the patient’s assessment and treatment plan.^3^

## Wheat Allergy

### Epidemiology

The epidemiology of wheat allergy is difficult to estimate due to the discrepancy between self-reported symptoms, serological sensitization, and food-challenge confirmation. A recent meta-analysis found the prevalence of wheat allergy to be 0.63% (95% CI: 0.43%-0.87%) for self-reported, 0.70% (95% CI: 0.18%-1.22%) for self-reported physician-diagnosed, 0.22% (95% CI: 0.07%-0.65%) for skin prick test positive, 0.97% (95% CI: 0.43%-2.20%) for specific immunoglobulin E positive, and 0.04% (95% CI: 0%-0.16%) for food challenge.^42^ The condition is most common in children, with the majority outgrowing the disease. A retrospective study on the development of wheat allergy found that rates of resolution were 29% by 4 years, 56% by 8 years, and 65% by 12 years. Higher wheat-specific immunoglobulin E (IgE) levels in blood were associated with poorer outcomes, and peak wheat-specific IgE was a useful predictor of sustained wheat allergy, although many children outgrew wheat allergy even with the highest levels of wheat-specific IgE.^43^

### Etiology

Whereas CD is primarily a Th1-mediated enteropathy in response to gluten,^44^ wheat allergy is primarily a Th2-mediated allergic response to various wheat proteins. α-amylase-trypsin inhibitor, a salt-soluble wheat protein, is one of the major allergens. It is implicated in baker’s asthma^45^ as well as anaphylaxis and wheat-dependent exercise-induced anaphylaxis (WDEIA).^46^ Due to its heat resistance, it is found in both raw and cooked wheat. In addition, salt-insoluble proteins, including ω-gliadins as well as low-molecular-weight glutenin subunits, are also able to bind IgE epitopes and are therefore implicated in wheat allergy.^47^

Wheat allergies are mediated by both IgE-mediated and non-IgE–mediated pathways. In the IgE-mediated pathway, the allergen induces crosslinking of IgE receptors on the mast cell surface, which in turn causes activation of mast cells and basophils. This activation results in the release of mediators such as histamine, platelet-activating factor, and leukotrienes, which in turn drive the allergic reaction.^4^ In the case of WDEIA, this is further precipitated by physical exercise. The non-IgE–mediated wheat allergies are mostly driven by eosinophilic infiltration in the gastrointestinal tract. Several food products have been implicated, with wheat being just one of them.^48^

### Clinical Manifestations

Clinical manifestations of IgE-mediated wheat allergy are diverse. Symptoms typically arise within a few minutes up to a maximum of 3 hours after exposure.^4^ The range of symptoms is diverse and can include several organ systems, including the gastrointestinal tract, circulatory system, airways, and skin. In patients with WDEIA, intense physical exercise is needed to trigger the anaphylactic symptoms, although some studies also indicate that other co-factors such as alcohol and non-steroidal anti-inflammatory drugs can trigger these reactions in the absence of exercise.^4^ Occupational exposure is associated with asthma or rhinitis in predisposed individuals, frequently referred to as baker’s asthma. Interestingly, this patient group often tolerates oral intake of wheat, indicating that the route of exposure plays a role in the clinical manifestation of the allergy.^49^ Regarding non-IgE–mediated wheat allergy, details regarding eosinophilic gastrointestinal diseases are beyond the scope of this review and are discussed elsewhere.^48^

### Diagnosis

The diagnosis of IgE-mediated wheat allergy can be difficult due to frequent discrepancies between serological sensitization and actual clinically manifest allergy. Patients can frequently display specific IgE against wheat; this does not always translate to clinically manifest allergy. This holds especially true in patients sensitive to grass (Timotei) pollen. Due to common IgE epitopes between grass pollen and wheat, there is frequent cross-reactivity.^4^ Wheat allergy is diagnosed with blood samples (increased serum specific IgE levels and components against wheat), a positive skin prick test with wheat, and/or provocation with wheat ingestion through oral open and/or placebo-controlled food challenges. In the case of suspected WDEIA, an oral provocation challenge can be performed under physical activity by a treadmill test.^4^ With regard to non-IgE–mediated allergies, eosinophilic gastrointestinal diseases usually require biopsies to confirm the diagnosis, followed by food elimination diets to confirm the allergen.^4^

### Management

The management of IgE-mediated wheat allergy centers around the avoidance of the triggering agents as well as managing adverse reactions in case of accidental exposure. In the event of anaphylaxis, epinephrine injections are the core treatment, supplemented with antihistamines, glucocorticoids, and β-agonists inhalations.^4^ Immunotherapy has shown promise as a treatment for wheat allergy, but has yet to be established in clinical practice.^4^ Non-IgE–mediated eosinophilic gastrointestinal diseases can be treated by avoidance of the triggering agent as well as drug therapy (for eosinophilic esophagitis: proton pump inhibitors, vonoprazan, and glucocorticoids; for eosinophilic gastroenteritis: antihistamines, leukotriene inhibitors, and glucocorticoids).^48^

## Non-Celiac Wheat Sensitivity

### Epidemiology

Investigations of the prevalence of NCWS have been hampered by a lack of uniformity as well as a high degree of self-reporting. In fact, the terminology itself remains heterogeneous. While the term “non-celiac gluten sensitivity” (NCGS) was initially proposed and popularized, it has been suggested that this could be renamed NCWS.^50^ This has implications beyond nomenclature; NCGS could include rye and barley due to similarities in prolamines found in wheat, rye, and barley. Non-celiac wheat sensitivity could potentially exclude rye and barley from the assessment. At the same time, the term NCWS is a recognition that the offending cereal protein is yet to be identified; it may, in fact, not be gluten, but rather another wheat protein.^50^ Both terms are currently used in the literature.

While self-reported prevalence of wheat intolerance ranges from 4% to 13%,^51^ a systematic review found that when subjected to double-blinded placebo-controlled trials, only 16% of such patients demonstrated gluten-related symptoms.^52^ It should be borne in mind that such reviews are limited by inter-study heterogeneity and should therefore be interpreted with caution.

### Etiology

There is no conclusive evidence regarding the pathophysiological mechanism behind NCWS. Some studies comparing immune responses in CD patients and NCWS patients indicate increased activity of the innate immune system, as seen in the Toll-like receptor 2, claudin-4, interleukin-8, and tumour necrosis factor alpha.^53,54^ This would indicate that the innate immune system plays a larger role in NCWS compared to CD, where the adaptive immune system plays a larger role.^53,54^ In fact, the very definition of NCWS as a distinct disease has been called into question due to its overlap with irritable bowel syndrome (IBS).

### Clinical Manifestations and Diagnosis

The most common symptoms associated with NCWS are irregular bowel movements, bloating, and abdominal pain. Eighty percent of suspected NCWS patients present with gastrointestinal symptoms such as bloating and abdominal pain.^55^ In addition, approximately 50% present with diarrhea and 20%-30% with alternating bowel habits, including constipation.^55^ These symptoms overlap significantly with IBS. Furthermore, non-specific extraintestinal symptoms ranging from fatigue to skin rash have been reported in 20%-30% of patients.^54,55^ There are currently no biomarkers or histological findings that have been verified for diagnostic purposes. Wheat allergy and CD must first be ruled out. The subsequent diagnostic process is based on the Salerno protocol.^50^ The first step is the assessment of symptomatic response to a gluten-free diet for at least 6 weeks. A lack of symptomatic response essentially rules out the condition. If there is a symptomatic response to a gluten-free diet, blinded placebo-controlled trials with gluten (alongside amylase-trypsin inhibitor) exposure and placebo controls are performed to confirm the diagnosis.

The potential overlap with IBS presents a diagnostic challenge. Wheat also contains fructans, which constitute part of the fermentable oligosaccharides, disaccharides, monosaccharides, and polyols (FODMAP) spectrum that can exacerbate IBS symptoms. A reduction in wheat intake simultaneously reduces FODMAP and gluten intake. Hence, symptomatic relief following avoidance of wheat could be interpreted as IBS responding to a reduction in FODMAP intake rather than a gluten/wheat intolerance. In fact, a double-blinded trial on patients with suspected NCGS found no evidence of specific or dose-dependent effects of gluten when the patients were on a low FODMAP diet.^56^ A subsequent double-blinded crossover study by Skodje et al^57^ exposed patients to fructans, gluten, or placebo separately. In this study, only fructans induced symptoms. Another study by Lied GA et al.has also performed a randomized, double-blind, placebo-controlled study on gluten challenge in suspected NCGS. Rather than single rounds of gluten/placebo exposure, the patients were instead exposed to 2 placebo and 2 gluten challenges interspaced by 3-day washout periods. Out of 20 included patients, only 4 were able to correctly identify gluten exposure. Furthermore, higher symptom severity following gluten exposure compared to placebo exposure was not seen.^58^ Taken in conjunction, these studies challenge the role of NCWS/NCGS as a separate disease entity distinct from IBS. Further studies are recommended. Beyond gluten and FODMAPs, the role of these proteins, such as amylase-trypsin inhibitor, is also yet to be determined.^59^

### Treatment

As is the case for CD and wheat allergy, the treatment for NCWS would be avoidance of the offending agent. However, whereas CD requires strict avoidance of gluten, tolerance can be assessed in NCWS on a case-by-case basis.^3^ Although guidelines do not specify whether a nutritionist needs to be involved, it is strongly recommended that a nutritionist (or physician with adequate training) be involved with patient education and follow-up.

## Conclusion

Given the increasing public attention toward wheat intolerance as well as the popularization of gluten-free diets, it is important that clinicians be aware of the spectrum of wheat-related disorders. CD and wheat allergy are well-recognized conditions; the pathophysiological basis for these conditions is well established, as is the benefit of avoidance of the triggering agent. Conversely, NCWS poses a challenge. The high self-reported prevalence does not correspond with double-blinded placebo-controlled trials. In addition, the potential overlap with IBS also challenges the understanding of NCWS as a distinct clinical condition. Further research is needed on this topic to better understand the pathophysiology as well as guide further treatment.

## Data Availability

The data that support the findings of this study are available on request from the corresponding author.
